# Social Orientation and Diabetes-Related Distress in Japanese and American Patients with Type 2 Diabetes

**DOI:** 10.1371/journal.pone.0109323

**Published:** 2014-10-15

**Authors:** Kaori Ikeda, Shimpei Fujimoto, Beth Morling, Shiho Ayano-Takahara, Andrew E. Carroll, Shin-ichi Harashima, Yukiko Uchida, Nobuya Inagaki

**Affiliations:** 1 Department of Diabetes, Endocrinology and Nutrition, Graduate School of Medicine, Kyoto University, Kyoto, Japan; 2 Department of Endocrinology, Metabolism and Nephrology, Kochi Medical School, Kochi University, Nankoku-shi Kochi, Japan; 3 Department of Psychology, University of Delaware, Newark, DE, United States of America; 4 Kokoro Research Center, Kyoto University, Kyoto, Japan; University of North Carolina at Chapel Hill, United States of America

## Abstract

**Objective:**

Recent evidence in cultural and social psychology suggests Eastern cultures' emphasis on harmony and connection with others and Western cultures' emphasis on self-direction and autonomy. In Eastern society, relational harmony is closely linked to people's well-being. The impact of this cultural and social orientation on diabetes-related distress was investigated.

**Research Design and Methods:**

Japanese and American patients with type 2 diabetes were surveyed by well-established questionnaire in Japan and in the United States, respectively. The association of personal values for interdependence, perceived emotional support, and the Problem Areas in Diabetes scale (PAID) were analyzed.

**Results:**

A positive correlation between interdependence and PAID (*r* = 0.18; *P* = 0.025) and a negative correlation between perceived emotional support and PAID (*r* = − 0.24; *P* = 0.004) were observed after adjustments for other factors in Japanese data (*n* = 149), but not in American data (*r* = 0.00; *P* = 0.990, *r* = 0.02; *P* = 0.917, respectively, *n* = 50). In Japanese data, the three-factor structure of PAID (negative feelings about total life with diabetes, about living conditions with diabetes, and about treatment of diabetes) was identified, and interdependence showed significant positive correlations with the first and second factors and perceived emotional support showed significant negative correlations with all three factors of PAID.

**Conclusions:**

These results suggest that personal values for interdependence may be linked to the level of diabetes-related distress and that the distress may be relieved by perception of emotional support, especially in an interdependent cultural context.

## Introduction

Successful diabetes care requires effective approaches to supporting behavior change of patients. Based on existing theories developed mainly in Western countries, a variety of behavioral and psychosocial interventions are implemented. Review of these interventions shows a philosophical foundation provided by “empowerment” of patients and a patient-centered approach enabling internal motivation to change [1]. Most theories emphasize changing patient behavior through the patient's own intention and ability [1]. The internally motivated “Losing weight is really important to me.” replaces the externally motivated “My doctor wants me to lose weight.” [2]. This concept is widely accepted and utilized by many certified diabetes educators [3]. However, external motivation may be more relevant in different cultural contexts.

Recent evidence in cultural and social psychology indicates that substantial cultural differences exist in a globalized world. In different cultural contexts, people exhibit different ways of thinking, feeling, and behaving. [4]. In the past two decades, a line of research using experimental methodologies has formed a theoretical framework comparing Western cultures (as exemplified by North American culture) with Eastern cultures (as exemplified by East Asian cultures) [5]. Western cultures are characterized by social orientation valuing “independence” or a model of agency that emphasizes self-direction and autonomy. In such cultures, people's internal attributes such as their own goals, desires and judgments form the predominant basis of their action [5]. This view is consistent with the existing theoretical foundation in diabetes intervention that focuses on the patients' internal motivation to health. In contrast, Eastern cultures such as China, Japan and Korea tend to place a higher value on “interdependence” or a model of agency emphasizing harmony, relatedness, and connection with others. In such cultures, people tend to act in consideration of the expectations, desires, and needs of others as the predominant basis of their action [5]. In these cultures, patients with diabetes often may focus on the potential deleterious effects of their lifestyle change on the people around them. Patients' perception of the expectations of others may have more impact on the ability to change their lifestyle in Eastern cultures than is found in Western cultures.

Indeed, results from social and cultural psychology indicate that these cultural differences lead to correspondingly divergent consequences in people's motivation and emotion. For example, North Americans are found to be more strongly motivated to maintain personal control while Japanese are found to be more strongly motivated toward relational harmony [6]. Attaining personal goals leads to enhanced well-being among European Americans while attainment of relational goals is more closely linked to enhanced well-being among Asian Americans and Japanese [7]. This emphasis on social relations is also shown in the result that East Asians' well-being is strongly predicted by social harmony, socially engaging emotions, and perceived emotional support from close others [8]. Perceived emotional support is a perception of receiving encouragement, compassion, and other forms of emotional support from the persons close to the respondent such as family members and friends. This result suggests that in an interdependent society people are more sensitive to the expectations of others. Similar results are shown in patients with diabetes. In Mexican Americans, who are characterized by a relatively higher tendency to interdependence than European Americans, social context reflected by the patients' perception that their family understands their diabetes is associated with patients' higher attention to self-care [5], [9], [10]. A family-centered approach in Taiwanese patients enhances patients' positive attitude toward diabetes [11].

The increasing rate of diabetes among Asians worldwide and Asian Americans calls for understanding the unique needs of diabetes care in Asian patients [12], [13]. One notable qualitative study suggested that interdependence and reciprocal role responsibility additionally complicate disease management in Chinese American patients with type 2 diabetes [14]. The interdependent cultural contexts of East Asian society may be an important way to conceptualize the unique needs of Asian patients with diabetes. However, not all members of a culture internalize its values to the same degree; people within a culture vary in their relative agreement with interdependent or independent values [15]. Therefore, it is important to test for within-culture associations of individual difference in interdependence/independence values and individual variation in coping with diabetes.

Psychosocial assessment is recommended in routine care of patients with diabetes; emotional well-being is associated with positive diabetes outcomes [16]. Diabetes-related distress is a psychosocial issue known to impact health outcomes: it is independently associated with self-management behaviors and perceived burden of diabetes and also predicts future glycemic control [17], [18]. Interdependent social orientation in Eastern cultures might therefore play a role in other psychosocial aspects of diabetes care. Strongly held personal values of interdependence may complicate diabetes care because changes in diet and lifestyle are magnified in such settings; patients who are interdependent may be more solicitous of their potential impact on others. On the other hand, perceptions of emotional support, encouragement and compassion from people around them may be especially effective for those in interdependent cultural contexts. Such emotional support may decrease the psychological burden of diabetes care on the patients personally.

In the current study, we explore differences in interdependence and perceived emotional support in relation to diabetes-related distress in two cultural backgrounds, Japan and the United States.

## Methods

### Participants

Participants were recruited from the Kyoto University hospital in Kyoto, Japan during the period of November 2009 through October 2010 and the Christiana Care Health System in Delaware, United States during the period of April 2010 through April 2012. Patients aged ≥30 years with type 2 diabetes for more than one year were eligible. Patients with depression were excluded from the following analysis.

### Procedure

Kyoto University Graduate School and Faculty of Medicine, Ethics Committee and the Institutional Review Board of Christiana Care Health System approved the study protocol. Participants were recruited at the diabetes outpatient clinic of each hospital. All participants provided written informed consent prior to participation. The survey measuring diabetes-related distress, interdependence, and perceived emotional support were completed by all participants. The participants then completed the sociodemographic questions (age, sex, education level, and occupational status). Years from diagnosis, treatment, history of attending a diabetes patient education program and presence of diabetes complications (retinopathy, nephropathy, neuropathy, stroke, coronary heart disease, and foot ulcer) or other comorbidities needing treatment or self-management such as hypertension, heart disease, malignant tumor, and depression, were also measured by a self-report checklist. Recent glycemic control (HbA1c) was obtained from medical records.

### Measurements

Diabetes related distress was measured using the Problem Areas in Diabetes scale (PAID), a well-validated 20-item self-report questionnaire [17]. Items are rated on a 5-point scale ranging from 0 (not a problem) to 4 (a serious problem). Summed scores are converted to a 0–100 scale by multiplying by 1.25 [19]. The PAID was translated into Japanese by Ishii et al. and the Japanese version also showed high internal consistency (Cronbach's α = 0.93) and validity [20]. Interdependence was measured by a well-established English and Japanese version of the Self-Construal Scale [21], [22]. Participants indicated how much they agreed with 10 independent statements (e.g., “I am not concerned if my ideas or behavior are different from those of other people”, “I do my own thing, regardless of what others think.”) and 10 interdependent statements about the self (e.g., “I am concerned about what people think of me”, “I often have the feeling that my relationships with others are more important than my own accomplishment.”). This scale has successfully measured independence and interdependence in many cultures including Japan and the United States. It distinguishes not only cultural variation but also individual variation in one culture. Measured scores are associated with psychopathological symptoms and neural activity in general population [23]–[25]. The score is the mean rating given to interdependent statements minus mean rating given to independent statements, which shows substantial internal reliability (split-half correlation = 0.53) [22]. Perceived emotional support was measured by a well-established English and Japanese language version of a 16-item scale assessing the perception of receiving encouragement, compassion, and other forms of emotional support from close others. Participants were asked to think about close others and then to indicate the extent to which these close others provided each of 16 types of emotional support (Cronbach's α = 0.91,.92, and.91, for Americans, Filipinos, and Japanese, respectively)[8]. Self-esteem was measured by Self-Competence scale, a well-established scale in English and Japanese [26], [27]. HbA1c measured in US was expressed according to National Glycohemoglobin Standardization Program (NGSP) and HbA1c measured in Japan was expressed as NGSP equivalent value [28].

### Data analysis

Participant characteristics and survey responses are presented as means and SD or sample size and percent. Distribution of variables was checked visually and by Shapiro-Wilk tests. Independent-sample t tests and Mann-Whitney U tests were used to explore group differences for normally distributed variables and for non-normally distributed variables, respectively. Fisher exact test was used for categorical data. Pearson's correlation coefficient was used to identify correlation among PAID, interdependence, perceived emotional support, self-esteem, and potential confounders such as sex, age and education level. The associations between PAID and interdependence and between PAID and perceived emotional support were assessed by two-way scatter plots and Pearson's correlation coefficient after adjusting for the identified confounders by regression model. To evaluate constructed factors of PAID that may have association with interdependence or perceived emotional support, the factor structure of PAID was analyzed in the Japanese data. In principal component analysis, an eigenvalue of>1.0 was used to identify the possible numbers of components. An exploratory factor analysis with promax rotation was performed. A loading level of> 0.40 was used for the items to be included in each component. The association between the identified factors of PAID and interdependence, perceived emotional support, self-esteem, sex, age, education level, HbA1c, years with diabetes, medications and complications were examined by multiple regression analyses. All analyses used Stata 11.0 (Stata Corporation, College Station, TX). Statistical significance was set at P<0.05 (2-tailed). Missing data were not imputed, with the exception of a maximum of 2 missing values of PAID, which were estimated using the mean of their remaining items [29].

## Results

Eligible participants who completed the surveys were 152 in Japan and 64 in the United States. All 152 participants recruited in Japan were of Japanese ethnicity. Of these, three were excluded from analyses because of missing data, two for occupation and one for education. The longest experience living abroad reported by a Japanese patient was four years. Only about 21 participants recruited in the United States during the period of September 2011 through April 2012 were able to confirm ethnicity; fifteen (71%) were European Americans, four (19%) were African Americans, and two (10%) were Asian Indians. Two of the 64 participants in the United States were excluded because of depression. Twelve were excluded because of missing data, one for HbA1c, one for education, five for interdependence and five for self-esteem. Of the remaining 50 participants, one reported that she had lived in Puerto Rico for 36 years, two of them had lived in India for 35 and 21 years respectively, one had lived in Ireland, England and Germany for 30 years total, one in India and Canada for 25 years total, one in England for 11 years, and others had lived abroad for no more than one year. In this study, these 50 participants comprised the American patients. Finally, 149 Japanese patients and 50 American patients were included in the analyses (Table 1). No significant differences were observed between the two groups in sex, age, education level, HbA1c, treatment, or diabetes education history. Japanese patients had lower BMI and slightly fewer years with diabetes than American patients. More American patients had nephropathy and neuropathy. Japanese patients had a higher score of interdependence and PAID and a lower score of perceived emotional support and self-esteem than American patients.

**Table 1:** Characteristics of patients.

	Japanese	American	*P*
*n*	149	50	
Female	58 (39)	25 (50)	0.187
Age (years)	60.6±8.6 (36∼81)	60.0±10.1 (33∼82)	0.655^†^
Education (years)	14.0±2.9 (9∼23)	14.6±2.4 (10∼21)	0.094^‡^
Occupation			
Full-time job	66 (44)	22 (44)	1.000
Part-time job	20 (13)	1 (2)	0.030
Without job or retired	63 (42)	27 (54)	0.189
BMI (kg/m^2^)	25.3±4.9 (15.1∼53.0)	32.6±6.5 (21.0∼51.4)*	<0.001^‡^
HbA1c (%)	7.6±1.2 (5.4∼11.2)	7.6±1.6 (5.6∼12.3)	0.285^‡^
HbA1c (mmol/mol)	60±13.1 (36∼99)	60±17.5 (38∼111)	
Years with diabetes (years)	10.1±8.4 (1∼38)	12.0±7.4 (2∼35)	0.030^‡^
Treatment			
Diet alone	21 (14)	2 (4)	0.072
OHA alone	82 (55)	25 (50)	0.623
Insulin alone	17 (11)	7 (14)	0.621
Insulin and OHA	29 (19)	16 (32)	0.079
Diabetes education history	74 (50)	27 (54)	0.627
Diabetes complication			
Retinopathy	24 (16)	3 (6)	0.094
Nephropathy	4 (3)	5 (10)	0.046
Neuropathy	14 (9)	11 (22)	0.027
Stroke	6 (4)	3 (6)	0.694
CHD	15 (10)	9 (18)	0.140
Foot ulcer	1 (1)	2 (4)	0.156
Major comorbidity			
Hypertention	20 (13)	8 (16)	0.643
Heart disease	10 (7)	5 (10)	0.535
Malignant tumor	1 (1)	3 (6)	0.050
Interdependence	− 0.06±0.84 (−2.7∼2.6)	− 0.52±1.20 (−2.9∼2.8)	0.001^‡^
PAID	29.8±18.7 (0∼92.5)	24.9±23.1 (0∼85)	0.030^‡^
Perceived emotional support	3.8±0.6 (1.9∼5.0)	4.3±0.6 (3.0∼5.0)	<0.001^‡^
Self-esteem	24.5±4.3 (14∼38)	28.5±6.0 (17∼40)	<0.001^†^

Data are n (%) or mean ± SD (range).

^*^ *n* = 47.

OHA, oral hypoglycemic agent; CHD, coronary heart disease; PAID, the Problem Areas in Diabetes scale.

P values are of group differences by independent-sample t tests for normally distributed variables^†^, Mann-Whitney U tests for nonnormally distributed variables^‡^, and Fisher exact test for categorical data.

**Figure 1:**
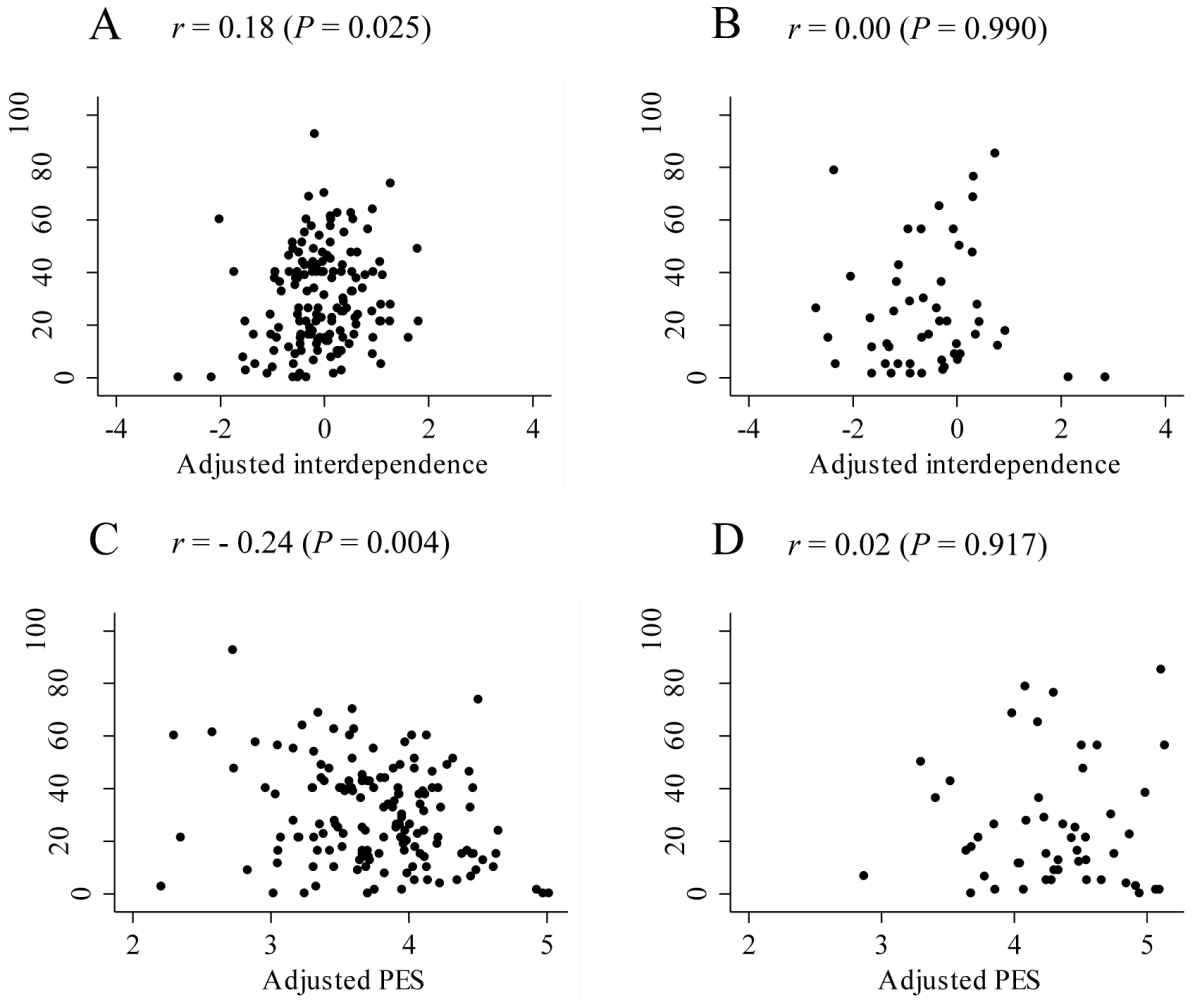
Distribution plots and Pearson's correlation coefficients to show the association between PAID and interdependence after adjusting for PES, self-esteem, sex, age and education level in Japanese (A) and Americans (B), between PAID and PES after adjusting for interdependence, self-esteem, sex, age and education level in Japanese (C) and Americans (D). PAID, the Problem Areas in Diabetes; PES, perceived emotional support.

In Pearson's correlation analysis, PAID had significant correlations with interdependence, perceived emotional support, self-esteem, sex and age in Japanese, and with self-esteem and age in Americans (Table 2). Interdependence had significant correlations with perceived emotional support, self-esteem, sex, age and education level in Japanese, and with self-esteem in Americans. Perceived emotional support had significant correlations with sex and age in Japanese, and with self-esteem in Americans. Based on these results, association between PAID and interdependence was assessed after adjusting for perceived emotional support, self-esteem, sex, age and education level. The association between PAID and perceived emotional support was also assessed after adjusting for interdependence, self-esteem, sex, age and education level. Adjusted interdependence showed a weak but significant positive association with PAID in Japanese (*n* = 149, *r* = 0.18, *P* = 0.025) (Fig. 1A), while it did not in Americans (*n* = 50, *r* = 0.00, *P* = 0.990) (Fig. 1B). Adjusted perceived emotional support showed a significant negative association with PAID in Japanese (*n* = 149, *r* = −0.24, *P* = 0.004) (Fig. 1C), but in Americans it had no association with PAID (*n* = 50, *r* = 0.02, *P*  = 0.917) (Fig. 1D). The two-way distributional patterns of adjusted perceived emotional support and PAID were strikingly different between Japanese and Americans. The more emotional support Japanese patients perceived, the less distress they reported. On the other hand, American patients who perceived more emotional support did not as frequently report less distress. Confining the analysis to patients without missing data of PAID did not influence the results. Confining the analysis to those who had not lived abroad more than 5 years also did not influence the results [30].

**Table 2:** Correlations between diabetes-related distress, interdependence and perceived emotional support.

	PAID	Interdependence	PES	Self-esteem	Sex	Age
Interdependence	0.28^‡^	—	—	—	—	—
	0.18	—	—	—	—	—
PES	− 0.17^*^	0.18^*^	—	—	—	—
	− 0.11	− 0.03	—	—	—	—
Self-esteem	− 0.30^‡^	− 0.39^‡^	0.04	—	—	—
	− 0.39^†^	− 0.42^†^	0.38^†^	—	—	—
Sex	− 0.22^†^	− 0.22^†^	− 0.31^‡^	0.24^†^	—	—
	− 0.18	− 0.11	0.07	0.17	—	—
Age	− 0.16^*^	− 0.17^*^	0.17^*^	0.26^†^	− 0.06	—
	− 0.31^*^	− 0.07	0.00	0.04	− 0.08	—
Education	− 0.13	− 0.21^†^	− 0.09	0.22^†^	0.31^‡^	− 0.15
	− 0.27	0.07	− 0.12	− 0.07	0.15	0.10

Pearson's coefficients (Upper: Japanese, Lower: Americans): ^*^ *P*<0.05; ^†^ *P*<0.01; ^‡^ *P*<0.001. PAID, the Problem Areas in Diabetes scale; PES, perceived emotional support; Sex, male = 1, female = 0.

In the Japanese patients, principal component analysis identified four factors of PAID with eigenvalue of 9.25, 1.36, 1.17, and 1.06. Each factor accounted for 46.3, 6.8, 5.8 and 5.3% of the variance, respectively. Assuming 2 to 4 factors, exploratory factor analysis was performed with promax rotation. In the 2 and 4 factor solutions, each factor was not homogeneous and hard to interpret. The conceptual congruency of items supported the 3 factors solution. The first factor included 8 items with loadings from 0.40 to 0.77, and could be interpreted as negative feelings about total life with diabetes (Table 3). The second consists of 8 items with loadings from 0.43 to 0.74, and could be interpreted as negative feelings about living conditions with diabetes. The third consists of 2 items with loadings of 0.75 and 0.77, and could be interpreted as negative feelings about treatment of diabetes. Cronbach's α as a measure of internal consistency for the 3 factors were 0.90, 0.84, and 0.82, respectively. The mean score of the items was calculated for each factor and used as a score of each subdimension of PAID. The score of the first subdimension, “negative feelings about total life with diabetes”, was 1.5±0.9 (mean±sd), and ranged 0 to 4. The second, “negative feelings about living conditions with diabetes”, was 0.8±0.7, and ranged 0 to 3.25. The third, “negative feelings about treatment of diabetes”, was 1.2±1.0, and ranged 0 to 4.

**Table 3:** Factor loadings of the 20 items of PAID for the three extracted factors after promax rotation in Japanese.

	Factor 1	Factor 2	Factor 3
Factor 1: negative feelings about total life with diabetes (α = 0.90)			
Feeling depressed when you think about living with diabetes	0.77	0.03	0.21
Feeling scared when you think about living with diabetes	0.72	− 0.20	0.43
Worrying about the future and the possibility of serious complications	0.57	0.04	0.13
Feeling angry when you think about living with diabetes	0.55	0.47	− 0.20
Feeling overwhelmed by your diabetes	0.55	0.32	0.01
Feeling constantly concerned about food and eating	0.45	0.23	0.12
Feelings of guilt or anxiety when you get off track with your diabetes management	0.40	0.35	0.01
Not “accepting” your diabetes	0.40	0.07	0.11
Factor 2: negative feelings about living conditions with diabetes (α = 0.84)			
Feeling alone with your diabetes	0.02	0.74	− 0.06
Feeling that your friends and family are not supportive of your diabetes management efforts	− 0.09	0.62	0.17
Feeling that diabetes is taking up too much of your mental and physical energy every day	0.24	0.55	0.02
Feeling “burned out” by the constant effort needed to manage diabetes	0.17	0.51	0.19
Not knowing if your mood or feelings are related to your diabetes	0.39	0.47	− 0.04
Feeling unsatisfied with your diabetes physician	− 0.18	0.47	0.20
Uncomfortable social situations related to your diabetes care (e.g., people telling you what to eat)	− 0.00	0.46	0.30
Worrying about low blood sugar reactions	0.23	0.43	− 0.20
Factor 3: negative feelings about treatment of diabetes (α = 0.82)			
Not having clear and concrete goals for your diabetes care	0.12	− 0.01	0.77
Feeling discouraged with your diabetes treatment plan	0.09	0.06	0.75
Unclassified items			
Feelings of deprivation regarding food and meals	0.19	0.39	0.13
Coping with complications of diabetes	0.30	0.12	0.38

PAID; the Problem Areas in Diabetes scale.

The association between the three subdimensions of PAID and interdependence and perceived emotional support was further evaluated by multiple regression analysis. As potential predictors of the identified three subdimensions, sex, age, education level, HbA1c, years with diabetes, medications and complications were considered to add to the three main predictors, which are interdependence, emotional support and self-esteem. Since age and education level were not significant predictors for all three subdimensions, they were removed from the model. “Negative feelings about total life with diabetes” was significantly associated with interdependence, perceived emotional support, self-esteem, sex, oral hypoglycemic agent, insulin and complications (Table 4). “Negative feelings about living conditions with diabetes” was significantly associated with interdependence, perceived emotional support, HbA1c and insulin. “Negative feelings about treatment of diabetes” was significantly associated with perceived emotional support and HbA1c.

**Table 4:** Standardized partial regression coefficients of potential predictors for the three PAID subdimensions in Japanese.

Predictor variables	Dependent variables		
	Negative feelings about total life with diabetes	Negative feelings about living conditions with diabetes	Negative feelings about treatment of diabetes
Interdependence	0.21 (0.009)	0.17 (0.047)	0.11 (0.215)
PES	− 0.19 (0.013)	− 0.21 (0.012)	− 0.23 (0.008)
Self-esteem	− 0.21 (0.008)	− 0.13 (0.121)	− 0.15 (0.091)
Sex	− 0.17 (0.030)	− 0.10 (0.245)	− 0.06 (0.481)
HbA1c	0.14 (0.062)	0.20 (0.012)	0.26 (0.002)
Years with diabetes	− 0.06 (0.388)	− 0.07 (0.386)	− 0.15 (0.064)
OHA	0.19 (0.012)	0.12 (0.126)	0.04 (0.598)
Insulin	0.16 (0.045)	0.19 (0.022)	0.05 (0.531)
Complications	− 0.17 (0.020)	− 0.12 (0.112)	− 0.09 (0.231)
Adjusted *R^2^* of overall model	0.28 (<0.001)	0.19 (<0.001)	0.15 (<0.001)

PAID, the Problem Areas in Diabetes scale; PES, perceived emotional support; Sex, male = 1, female = 0; OHA, oral hypoglycemic agent, use = 1, nonuse = 0; Insulin, use = 1, nonuse = 0; Complications, if any = 1, none = 0.

Data are standardized partial regression coefficients of each predictor with *P* values in parenthesis and adjusted *R^2^* of overall model with *P* values in parenthesis.

Age and education level were removed from the models because they were insignificant predictors for all three subdimensions.

## Discussion

We investigated the contribution of social orientation emphasizing harmonious relations with others to diabetes-related distress in Japanese and American patients. The results indicate that a patients' tendency to interdependence may increase diabetes-related distress, and that a perception of encouragement and compassion from people around them may decrease the distress especially in Japanese patients living in an interdependently oriented society. In the current study, Japanese patients with higher personal values for interdependence reported higher levels of distress, and Japanese patients who perceived more emotional support reported lower levels of distress.

Such cross-sectional correlations need to be cautiously interpreted. An interdependent social orientation is reflected in one's generalized pattern of thought, feeling, and action [5], [15]; among patients who value harmonious relations with the people around them, diabetes self-care requires adjustments to relationships that will add additional distress to the patients. Japanese patients who are interdependent may be especially conscious of, and concerned about, the impact of their required lifestyle changes on close others.

Notably, the association between perceived emotional support and diabetes-related distress observed in Japanese patients was not observed at all in American patients. Although the perceived emotional support addressed in the survey was general and not specific for diabetes, there was a significant negative association with diabetes-related distress. This suggests that patients' feelings that close others encourage and empathize with them may play an especially important role in diabetes self-care in a highly interdependent culture. This finding replicates past studies in which perceived emotional support was positively related to subjective well-being among Filipinos and Japanese, but not among Americans [8].

Japanese patients with higher interdependence were more likely to experience distress related to life with diabetes than to treatment of diabetes. This seems reasonable considering that interdependence affects total life and living conditions with diabetes but not treatment of diabetes. In addition, higher self-esteem may be effective in reducing the distress related to total life with diabetes, but the effect of self-esteem was smaller and was not related to the distress related to living conditions with diabetes and treatment of diabetes. Self-esteem measured in this study was confidence in one's ability not specific for diabetes. The result indicates that Japanese patients with high confidence in their general ability may be relatively resistant to distress related to total life with diabetes, but that their tendency to interdependence may increase the distress related to total life with diabetes.

Perceived emotional support had a general positive effect in all three subdimensions of distress. In an interdependent society such as Japan, encouragement and compassion from people around them may have a wide range of effects on diabetes-related distress. Among other potential contributors to diabetes-related distress, male sex was significantly associated with a lower level of distress about total life with diabetes, and poor control was significantly associated with higher level of distress about living conditions with diabetes and treatment with diabetes. Duration of diabetes was not a significant contributor to diabetes-related distress in this study. These results accord with a previous report [18], [19], [29].

Patients treated by medication had a higher level of distress related to total life and living conditions with diabetes but not to treatment. This result suggests that negative feelings about treatment do not necessarily stem from the medication itself. However, the result also suggests that medication itself may nevertheless be an important factor in increasing distress related to total life and living conditions with diabetes. We also found a counterintuitive association between diabetes complications and distress. Patients without any diabetes complications showed a higher level of distress related to total life with diabetes. One possible explanation is that patients without any complications were more anxious about developing complications than were those already having one or more complications. In this subdimension analysis, we used only Japanese data because of the small sample size of American data for factor analysis. Previously reported factor structures of PAID vary from study to study, and one to four factors are identified. Our results are relatively similar to the results in Dutch and Swedish patients [29], [31].

We used well-established measures of interdependence, perceived emotional support, and diabetes-related distress and a growing body of evidence [8], [17], [20−22], [26], [27], . Our study found that emotional well-being of patients with diabetes was predicted by different variables in Japan than in the United States. Distress caused by diabetes self-care was influenced by both individual and cultural variation of interdependence. Cultural psychology takes the view that human cognitive and affective processes vary as a function of cultural environments that provide unique social contexts in which psychological processes develop and are shaped [4], [32]. Although past research has shown that independence and interdependence are associated with a variety of daily behaviors in healthy people, the unique contribution of the present study is that it studies these cultural variables in a specific health context. Our work thus replicates and extends past work on cultural patterns to the diabetes context.

A similar comparison may be applicable to other societies and cultures; a cultural emphasis on interdependence is also known to be a characteristic of many African cultures, Latin-American cultures, and many southern European cultures [5]. Asian-American cultures within the United States are reported to similarly emphasize interdependence, although the magnitude of this difference may be smaller than in those living in Asian countries [5]. The findings in this study provide better understanding of the differences between European-American patients with diabetes and other patients. This study focuses on the difference between two cultural contexts: Japanese patients recruited in Japan and American patients recruited in the United States. It enables us to interpret the results without the influence of acculturation or linguistic barrier for minority. An important limitation of this study is the small sample size of the American patients. Further study is required to investigate the impact of individual variation of interdependence on diabetes-related distress among American patients.

This study suggests a potential link between interdependent social orientation and various outcomes of diabetes care. Interventions appropriate for interdependent social orientation are required. Family-centered approaches may be an effective option in such interdependent patients.

## Supporting Information

Dataset S1 (DTA)
